# Research Progress in Intelligent Monitoring of Livestock, Poultry, and Aquaculture

**DOI:** 10.3390/ani16071033

**Published:** 2026-03-27

**Authors:** Yueju Xue, Haiming Gan

**Affiliations:** College of Electronic Engineering, South China Agricultural University, Guangzhou 510642, China

## 1. Introduction

With the rapid advancement of intelligent farming technologies, precise monitoring, efficient management, and animal welfare assurance have become the core demands of industrial development. These two Special Issues collectively include 16 research articles focusing on livestock, poultry, and aquaculture scenarios. Drawing on interdisciplinary perspectives from computer vision, biomechanics, and physiology, these studies address key tasks including behavior recognition, individual monitoring, health assessment, and population counting. Collectively, the contributions presented in these two Special Issues demonstrate a full-chain innovation pathway from algorithmic development in laboratory environments to practical deployment in real farming conditions. The studies not only highlight the practical value of technological breakthroughs but also emphasize the animal-centered welfare orientation. Together, they provide both theoretical insights and technical frameworks for the standardized and scalable development of intelligent livestock and aquaculture systems.

## 2. Overview

The sixteen studies included in these two Special Issues predominantly rely on deep learning-based methodologies and propose targeted architectural optimizations to address the complex and heterogeneous conditions of farming environments.

In duck farming scenarios, Zhao et al. (Contribution 1) proposed a posture estimation approach based on HRNet-32-CBAM, capable of detecting eight key points of ducks and correctly linking them to estimate six behavioral postures. This research contributes to welfare breeding and abnormality monitoring of ducks. Duan et al. (Contribution 2) proposed an activeness estimation method for group-housed ducks using a multi-object tracking model, enabling the measurement of duck activeness and investigation of the influence of photoperiod on duck activeness. The proposed method and conclusions regarding the optimal photoperiod provide both methodological and data support for enhancing duck breeding and farming efficiency. To address monitoring challenges in dense duck houses, Xie et al. (Contribution 3) developed an integrated detection and tracking framework that achieves real-time, high-accuracy detection and stable multi-object tracking. The system enables reliable duck monitoring and counting in crowded environments and offers a practical, low-cost solution for intelligent duck farming.

In pig farming scenarios, Luo et al. (Contribution 4) proposed a novel method based on the improved YOLOV5 and feeding functional area suggestions to identify the feeding behaviors of nursery piglets in a complex light and different posture environment. The method enables automated and continuous monitoring of pig feeding behavior, helping producers optimize feeding strategies, reduce feed waste, and improve production efficiency. Subsequently, Luo et al. (Contribution 5) developed a lightweight behavior detection model based on the YOLOv8s architecture, capable of real-time recognition of eight core piglet behaviors. Through the coordinated use of model pruning and knowledge distillation, the approach achieves a balance between model compression and recognition accuracy, enabling deployment under limited computational resources while supporting welfare-oriented behavioral monitoring. Addressing the issue of lost ear tags in breeding pigs, Duan et al. (Contribution 6) proposed a dual-view collaborative detection framework integrating top-view and panoramic oblique-view cameras through a detection–mapping–tracking pipeline. This method effectively identifies untagged pigs and reduces identity loss caused by missing ear tags, thereby improving farm management efficiency. Beyond behavior monitoring, several studies focus on pig health assessment and welfare evaluation. Tu et al. (Contribution 7) developed Pig-ByteTrack, an automated framework for monitoring group-housed pigs that integrates target detection, multi-object tracking, and behavioral time analysis. The system enables farm managers to detect abnormal behaviors and potential health issues in a timely manner. In subsequent work, Tu et al. (Contribution 8) proposed a comprehensive pig health assessment framework based on multi-object behavior tracking and analysis under large-scale pig farming. By automatically tracking and quantifying four major pig behaviors in commercial farms, the framework evaluates behavioral health status and provides effective technical support for modern pig health monitoring. To facilitate physiological monitoring, Ma et al. (Contribution 9) constructed TIRPigEar, a thermal infrared dataset of pig ears. The dataset was validated using state-of-the-art deep learning models and provides a valuable resource for improving pig condition monitoring and enabling early intervention in health management. To enhance cross-scene adaptability of pig tracking systems, Liu et al. (Contribution 10) proposed SDGTrack, a multi-object tracking model incorporating an adaptive module and improved tracking strategies. The model learns background information from different environments, improving cross-scene generalization and tracking robustness.

In aquaculture applications, Li et al. (Contribution 11) proposed the Penaeus Larvae Counting Strategy (PLCS). The method treats two different types of keypoints as equip keypoints based on keypoint regression to determine the number of shrimp larvae in the image. This method provides an efficient and non-destructive solution for shrimp seedling trading. In further work, Li et al. (Contribution 12) developed the deconvolution enhancement keypoint network (DEKNet), which performs fish fry counting using a single keypoint feature. The method effectively counts fish larvae even under overlapping and blurred conditions, offering a precise and efficient counting solution for intelligent aquaculture systems.

In other animal scenes, Wang et al. (Contribution 13) proposed GSCW-YOLO, a multi-scale and lightweight behavior recognition model for dairy goats capable of real-time processing and accurate detection of abnormal behaviors, providing technological support for intelligent management and welfare-focused breeding of dairy goats. In order to monitor and manage the giraffes in the zoo scientifically, Gan et al. (Contribution 14) proposed a multi-behavior automatic detection method for giraffes based on YOLO11-Pose and the spatial-adaptive two-stream network (SATSN). The method achieves high accuracy and stability in detecting giraffe daily behaviors. In the context of equestrian training, Schampheleer et al. (Contribution 15) explored horse gait classification using rider-mounted accelerometers. Their study systematically evaluated the effects of sensor placement, sampling frequency, time window size, and model type on classification performance, offering a non-invasive and high-precision solution for equestrian training optimization and horse welfare monitoring. For multi-species and multi-scenario tracking, Yin et al. (Contribution 16) proposed a correlation-filter-based animal tracking method (RFCFT) that achieves robust animal trajectory tracking through a correlation filter framework with response map analysis and adaptive feature updating. The method is efficient and accurate in tracking animals in complex scenes, providing essential basic data for animal behavior analysis.

## 3. Conclusions

The studies presented in these two Special Issues address practical challenges in modern animal production systems, including high-density housing, complex environments, long-term tracking, and stress-free monitoring. Significant progress has been achieved in object counting, behavior recognition, pose estimation, health assessment, individual tracking, and dataset construction. Collectively, these contributions establish a lightweight, efficient, non-invasive, robust, and deployable technological framework that supports animal welfare evaluation, early health warning, and improved farming efficiency.

From a broader perspective, three notable characteristics emerge across the contributions: Non-contact monitoring approaches, such as vision-based sensing and indirect wearable technologies, are widely adopted to minimize animal stress and disturbance. Targeted algorithmic improvements are proposed to address real-world challenges, including occlusion, crowding, low illumination, long-distance observation, and small-object detection. Increasing attention is given to model lightweighting, low-power consumption, and edge deployment.

Despite these advances, several common challenges remain. Performance degradation can still occur under extreme occlusion, ultra-high density, and cross-scene generalization scenarios. Furthermore, existing datasets still have room for expansion in terms of species diversity, age coverage, and environmental complexity. Continued validation is also needed to ensure the effective integration of these technologies into real production systems.

Looking forward, key technological directions include lightweight model deployment, multimodal data fusion, and edge-device adaptation. From an application perspective, future research is expected to move from single-function monitoring toward integrated multi-functional systems, from single-species solutions toward multi-species adaptability, and from laboratory prototypes toward large-scale real-world deployment.

